# “Someone will come in and say I'm doing it wrong.” The perspectives of fathers with learning disabilities in England

**DOI:** 10.1111/bld.12351

**Published:** 2020-10-29

**Authors:** Jon Symonds, David Abbott, Daryl Dugdale

**Affiliations:** ^1^ School for Policy Studies University of Bristol Bristol UK

**Keywords:** family, family support, gender, learning (intellectual) disabilities, parenting, parents with learning disabilities

## Abstract

- When people with learning disabilities have children and become parents, they sometimes need good support to help them.
- Research about parents with learning disabilities and the support they get is usually about mothers and not fathers. There is not very much written about dads with learning disabilities.
- This paper is about interviews with eight dads with learning disabilities who told us about what it was like to be a dad and about the kind of support they had got.
- We think more support needs to be given to parents with learning disabilities and that dads should be included in this.

**Abstract:**

**Background**

This article reports on the perspectives of fathers with a learning disability in England about being fathers and the support they have received. Although there is an established literature that considers parenting with a learning disability, few studies have focused on the perspectives of fathers.

**Method**

We adopted a qualitative approach for this study, using semi‐structured interviews with eight fathers with learning disabilities, recruited through learning disability organisations and social media. The data were analysed using thematic analysis.

**Results**

Being a father was important to participants and the findings are presented in three themes: descriptions of fatherhood; challenges of fatherhood; and support with fatherhood.

**Conclusion**

We discuss how fathers' perspectives on fatherhood relate to wider transformations of fatherhood in society, the impact of these on fathering with a learning disability and the implications for services.

## 1 INTRODUCTION

This article contributes to the literature on parenting with a learning disability by focusing on the perspectives of fathers. It presents the findings from interviews with eight fathers, drawn from a larger qualitative study conducted in England between 2015 and 2016 which interviewed eight men who identified as a father with a learning disability and nine practitioners from learning disability services. By hearing the voices of fathers, practitioners and policymakers can understand how best to support all parents with learning disabilities, so that their children can achieve their potential.

In recent decades, there has grown an established literature on the lives and needs of parents with a learning disability which is broad in range and international in scope (McConnell et al., 2017). Unsurprisingly, having a learning disability has been shown not to be predictive of parenting capacity (Powell & Parish, 2017; Tymchuck, 1992) and systematic reviews of the literature have concluded that when there is appropriate support, parents with learning disabilities are able to develop parenting skills such as giving feedback to children (Wilson et al., 2014), improve the safety of the home environment and recognise signs of illness in their child more effectively (Coren et al., 2011). When support is provided in a flexible way, adults with learning disabilities can adapt their parenting skills to meet their children's changing needs (Tarleton et al., 2006) and delivering parenting support in the home means that support can be tailored to that parent's particular circumstances (Wade et al., 2008). In a study of stress in mothers with learning disabilities, Stenfert Kroese et al. (2002) found that increasing social support was correlated with parental well‐being and reduced stress with the implication that when support is provided in an effective way, there are benefits for parents with learning disabilities as well as their children.

In the UK, this emphasis on support has been increasingly visible in government policy and guidance for services. The needs of parents with learning disabilities were recognised in the White Paper, Valuing People (Department of Health, 2001), followed by the recognition of the right of all disabled people to be parents in the United Nations Convention on the Rights of Persons with Disabilities (United Nations, 2006) and of adults with learning disabilities specifically (HM Government, 2009). Good practice guidance on supporting parents with learning disabilities was issued in 2007 (Department for Education & Skills & Department of Health, 2007) and this was updated in 2016 to include additional relevant case law (Working Together with Parents Network, 2016).

A limitation of the current literature is what Theodore et al. (2018) described as “sampling issues” with the research focusing almost exclusively on mothers. This has long been recognised in the field. In a practitioner review from 1993, Booth and Booth cited Llewellyn's observation from 1990 that “virtually” all information related to mothers and that fathers had been “neglected” (Booth & Booth, 1993) and the point has been reiterated in subsequent reviews. Collings and Llewellyn (2012) noted that the literature that “addresses fathers solely” was “severely limited” and the situation remained unchanged in 2017 when McConnell et al. (2017) wrote that fathers with learning disabilities remained “understudied.”

Although studies are increasingly including both fathers and mothers in their samples, it is common for fathers to represent only very small numbers of participants, resulting in their perspectives being less visible in the findings. For example, the systematic reviews cited above had combined samples of 200 mothers and 19 fathers, with each set of authors recommending this be addressed in future research (Coren et al., 2011; Wilson et al., 2014). This is important because fathers might relate to their identity as parents in different ways than mothers (Shewan et al., 2012) and may also have different experiences of services (Theodore et al., 2018). There may also be important differences between fathers from different backgrounds. O'Hara and Martin (2003) investigated a single English learning disability service and found clear differences between White English and Bangladeshi fathers, for example whether they were living with their children.

Understanding the particularities of the lives and needs of fathers with learning disabilities might therefore enable practitioners and policymakers to support them in more tailored ways in their parenting role. There is now widespread international consensus that when fathers in the general population are involved with their child through playing with them, reading to them and being involved in decisions about them, this is associated with reduced behaviour problems in boys, reduced psychological problems in girls and, for children in families with low socio‐economic status, reduced economic disadvantage in adulthood (Sarkadi et al., 2008). Scholars from a range of disciplines have worked to make sense of the position of fathers in families (Schoppe‐Sullivan & Fagan, 2020) as notions of what constitutes a “good father” have been debated. For example, in their sociolegal study of fatherhood, Collier and Sheldon (2008) outlined the shifts in legal constructions of fatherhood over history, from a position of entitled authority in the family, to a functionalist role of providing for the family through paid employment, and more recently to a notion of the “involved” father, in which fathers are encouraged to provide practical and emotional care to their children. Pleck's (2010) model of “involved” fatherhood conceptualises it by five components: positive engagement (such as care and play); warmth and responsiveness (including emotional support); control (such as setting rules and monitoring behaviour); indirect care (such as arranging health and education services); and process responsibility (monitoring and anticipating what tasks need to be done). Such a framework facilitates a more nuanced understanding of fathers' different practices in their children's lives which may be applicable to fathers in different contexts.

Despite the extensive range of literature on fatherhood, there has been very little that focuses specifically on fathers with learning disabilities. Although studies often include fathers in their samples, and some publications address both fathers and mothers from separate studies (Mayes & Sigurjonsdottir, 2010), we were motivated to find publications that focused solely on fathers with learning disabilities, and in March 2018, we searched for literature in CINAHL, IBSS, PsycInfo and Web of Science, using the search terms father* AND (learn* OR intellect* OR mental* OR cognit*) AND (disab* OR difficult* OR impair* OR handicap* OR retard*) with no date restriction. We reviewed 3,716 records and found only four peer‐reviewed publications that focused specifically on fathers, based on a larger survey in the United States (Peck & Stephens, 1965); a personal authored account from Australia (Strike & McConnell, 2002); an ethnographic study in Iceland (Sigurjonsdottir, 2004) and a focus group of three fathers in Scotland (Gosden & Kirkland, 2008). The studies span a period in history that witnessed a transformation in society's perspectives both on parents with learning disabilities and on the position of fathers in families and this is reflected in the findings of the studies. For example, in the 1960s, Peck and Stephens concluded that men with learning disabilities did not have the capacity to care for their children (Peck & Stephens, 1965), whereas by the turn of the century, fathers were found to be providing for their family through employment (Sigurjonsdottir, 2004) and speaking about the importance of taking a more involved “hands‐on” approach to fatherhood (Gosden & Kirkland, 2008; Strike & McConnell, 2002). Where studies reported on fathers' experiences of services, fathers spoke of feeling “side‐lined” (Sigurjonsdottir, 2004) and “excluded” (Gosden & Kirkland, 2008) with recommendations that services should take a gender‐informed approach to supporting parents with learning disabilities. Further searching in 2019 also identified some conference presentations (Adolfsson et al., 2019; Badran et al., 2019; Cwirynkalo, 2019; Mirfin‐Veitch, 2006; Wade et al., 2012) but we were not able to locate published articles from these. Given that parents with learning disabilities have long been over‐represented in represented in the child protection and care systems of the UK (Booth et al., 2005), Australia (Llewellyn et al., 2003), Canada (McConnell et al., 2011) and the United States (LaLiberte et al., 2017), we argue that it is important to better understand the experiences of fathers with learning disabilities so that practitioners might find better ways to support children to grow up well in their own family environment.

## 2 METHODS

The aim of this study was to understand the perspectives of fathers with a learning disability on being a father and the support they had received. The study was conducted in England and was funded by the School for Social Care Research to support the development of early career researchers. Symonds and Dugdale had each recently completed doctoral theses related to fathers and social work and had practice experience in working with adults with learning disabilities. They were mentored in the design and conduct of the study by Abbott who has extensive academic experience in the learning disabilities field.

Due to the limited research literature on the topic, the study design was exploratory and small in scale and funding. We adopted a gendered approach, drawing on Connell's (2005) theoretical framework of masculinities as multiple and contested within social spaces, but organised by hegemonic ideals of men as powerful, independent, successful and in control. Wilson et al. (2012) argued that this has particular implications for research in intellectual disabilities where there are competing discourses of hegemonic masculinity and impairment, frequently associated with deficit and dependency. Subsequent work by Abbott (2013) has shown how men with learning disabilities challenge this by negotiating agency and independence in their relationships, but the tension has particular implications for research into fatherhood, where changing notions of being a good father may provide additional resources for constructing identities as fathers. For example, the provider model of fatherhood is predicated on paid employment, but the model of the involved father includes childcare‐related tasks and is accessible to men who are not in paid employment, something which we hypothesised may apply to some men with learning disabilities.

We adopted an interpretivist methodological position using a qualitative design of semi‐structured interviews. These were preferred to a focus group because they offer participants opportunities to develop their responses without being interrupted and enable quieter, or less articulate participants to have their voice heard (Brinkmann & Kvale, 2015). We aimed to speak to ten fathers who identified as having a learning disability and ten from practitioners from learning disability services, and in this article, we report on the interviews with fathers. The interview topic guides included questions about what participants found easy and difficult about being a man, as a father, and what their experiences had been of support services.

We did not have the resources to work in a fully co‐produced way, but by working with an advocacy organisation, we incorporated the views of four fathers with learning disabilities at each stage of the study. Following the different levels of participation described by Arnstein (1969), we describe these fathers as consultants rather than partners because they gave views on ideas we had already developed, rather than planning and conducting the study together. As a result of their feedback, we strengthened the feasibility of the study, amended participation information sheets and consent forms for acceptability and included additional questions to the interview topic guide. We shared our analysis with the group for acceptability of the findings and worked with them on dissemination, including a short film about the findings (Moore & Lavan, 2017), and co‐delivering a presentation to a national conference on social work.

Letters of introduction, participant information sheets and consent forms were prepared in Plain English and Easy Read formats and ethical approval was granted by the National Social Care Research Ethics Committee. Information about the study was disseminated via advocacy and self‐advocacy organisations, social media and the Working Together with Parents Network, a network of practitioners who work with parents with a learning disability in England. We recognise the debates on terminology in this field and used the term “learning disability” in the study as it is the preferred term of Learning Disability England, a network of user‐led organisations in the field (Learning Disability England, 2019). A “learning disability” is defined in Valuing People as a combination of impaired intelligence and impaired social functioning which started before adulthood (Department of Health, 2001), but rather than ask participants to identify whether they met this definition, we considered participants eligible if they self‐identified as a person with a learning disability or if they had experience of learning disability services, as well as experience as a father (including being a stepfather).

### 2.1 Participants

Ten fathers with learning disabilities expressed interest in the study, five directly themselves and five through a supporter. They lived in different parts of England and after further information was provided by post and on the telephone, interviews were conducted at a location of their choice, with the option of having a supporter there if they wished (one father chose to have this). Two participants had to cancel their interviews and despite attempts to rearrange these, time constraints meant that they eventually withdrew from the study.

The participants ranged in age between 26 and 61, with a mean age of 42. Six of the participants identified as White British with one identifying as Black British and one Pakistani British. The earliest a participant had become a father was in the early 1980s, and there was therefore a range of perspectives from different points in history. The ages of their children ranged from 16 months to 34 years and they had all had some opportunity to practise being a father, although the amount of time which they did this was very different among the group. For example, Peter had six sessions of supervised contact with his son before being placed for adoption, while Joe brought up his children to adulthood. Although two participants had also become stepfathers, all participants were birth fathers who had been in a relationship with the mother at the time of their children's birth. Two fathers were also grandfathers themselves and one was a step‐grandfather. At the time the interviews took place, the participants were evenly split as to whether or not they were currently living with their children. The key characteristics of the eight fathers who took part are included in Table 1:

**TABLE 1:** Characteristics of participants

Name	Ethnicity	Age	Living situation	Living with child	Age of children	Occupation
Nick	White British	34	Living independently with partner and daughter	Yes	16 months	Advocate and trainer
Peter	White British	26	Supported living with two unrelated adults	No	2	Voluntary worker at farm
John	White British	38	Supported living with wife and daughter	Yes	3	Supermarket employee
Joe	White British	61	Living independently with wife and foster children	Yes	34 and not reported	Founder of user‐led organisation
Matt	Black British	47	Living with second wife	No	Four children between 16 and 27, and six stepchildren	Member of user‐led organisation
Mustafa	British Pakistani	34	Supported accommodation with 12 other adults	No	14, 10, 5	Member of user‐led organisation
Darren	White British	53	Living independently with wife and two sons	Yes	21, 17	Advocate and trainer
Simon	White British	43	Supported living	No	24, 22, 14	Advocate and trainer

Participants varied considerably in their levels of articulacy, and this affected the length of the interviews and the amount of data from each one, a feature noted by Kaspar and Stenfert Kroese (2017) in their study with mothers with learning disabilities. Six participants we spoke to were involved in self‐advocacy organisations, including two who were senior representatives of national organisations. They appeared more comfortable in interview situations and their responses were informed by knowledge of gender dynamics and government policy, whereas other participants were less so. As a result, we do not claim that our findings are representative of fathers with learning disabilities in general, but that they remain valid representations of the views of the fathers we spoke to.

## 3 DATA ANALYSIS

The interviews were audio‐recorded and transcribed with the names of all people, places and services anonymised to fictional alternatives. Symonds and Dugdale read the transcripts in detail and analysed them according to the principles of thematic analysis (Braun & Clarke, 2006), identifying initial codes, discussing and refining these, and organising them into subthemes and themes. Critiques of interview studies have highlighted the risks of claiming unmediated access to participants' interior experience without recognising the interactional context in which the interview data were produced (Atkinson & Silverman, 1997; Silverman, 2017). For example, Hollomotz (2017) identified some of the interactional problems that may occur when conducting interviews with adults with learning disabilities, such as “acquiescence” when a participant apparently agrees with the implication of the question. However, conversation analysts (Antaki & Crompton, 2015; Antaki et al., 2015) have shown that adults with learning disabilities demonstrate more agency than “acquiescing” suggests and respond to interactional cues. In our own analysis, we followed Hollomtz's suggestion to remove data before analysis, such as a participant responding “yeah, yeah” to the interviewer's suggestion that the housework was divided in a “fair, fairly even” way. We have also followed Potter and Hepburn's (2012) recommendation to include interviewers' utterances to provide context for participants' responses. The themes are presented in Table 2:

**TABLE 2:** Thematic analysis of interviews with participants

Theme	Subthemes
Descriptions of fatherhood	Hands‐on fathering; domestic duties and fathering; being a father in public; participants' own fathers
Challenges of fatherhood	Caring for children; negotiating with partner; partners and family; separation; poor mental health
Support with fatherhood	Help from family; help from services; feeling left out

## 4 FINDINGS

The findings from the data are presented in three themes: descriptions of fatherhood; challenges of fatherhood; and support with fatherhood.

### 4.1 Descriptions of fatherhood

#### 4.1.1 Hands‐on fathering

Most participants told us examples of “hands‐on” fathering related to the direct care of, or playing with, their children. There were many examples given in our data, often related to times when the children were babies; for example, Darren explained how he felt about feeding his son and changing his nappies:

Interviewer: What did you find the most enjoyable part, as a dad?

Darren: Feeding him

Interviewer: Feeding him

Darren: The worst part was changing nappies. I hate that, yeah. (Darren, 53 yrs)

There were fewer examples of caring for older children, because not all participants had this experience, but Simon described the importance of taking his children to school.

Interviewer: Right. And did you feel – how did you feel about that?

Simon: I felt happy, I felt like a proper parent taking my kids to school. I enjoyed it, talking to other parents, talking to other fathers, talking to other mothers. I liked it. (Simon, 43 yrs)

As well as direct care, participants gave examples of interacting with their children through play, whether this was Nick playing with his toddler daughter and “making funny faces,” or John using playdough with his three‐year‐old son. Participants with older children spoke of taking them on trips to the local area:

Interviewer: What sorts of things did you get up to, would you do?

Mustafa: We used to play with kids together, take them out to park, take them to library, take them to shopping. (Mustafa, 34 yrs)

Regardless of the types of activity, all participants described aspects of fatherhood as related to playing, or providing direct care. Peter's son was placed for adoption as a baby, but even with a small number of contact sessions, he described his experience of changing a nappy. Providing care and playing with children appeared to be a key means by which participants related to the notion of being a father.

#### 4.1.2 Domestic duties and fathering

In contrast to the hands‐on dimension of fatherhood, participants gave different accounts of their involvement in domestic chores. Joe explained when he was a single father, he cleaned the house, did the ironing and learned to sew, and Simon articulated the importance for him of men doing housework:

Simon: It takes two to make a child. I'll do my part, I'll look after my house. If I move in with a lady I will do my part of the housework and everything, not always do – not let a woman do it. That is sexist and old‐fashioned. (Simon, 43 yrs).

Other participants agreed that both parents would be involved in the housework, but their commitment to the parenting relationship was demonstrated through supporting their partner who took lead responsibility in this area. Darren summed this up succinctly:

Interviewer: And what about the housework and the cleaning and the tidying?

Darren: I help her. (Darren, 53 yrs)

Darren's response suggests a willingness to contribute to these areas, but that the primary responsibility for these tasks rested with his partner. This position was echoed by Nick who acknowledged that it was important to take turns, but that his work effectively meant that he expected his partner, Liz, to do most of the cleaning:

Nick: I say to Liz, we just need to keep the house ni‐ the flat nicely tidy and clean. As long as that's clean, I'm happy. And I don't expect Liz to do all of that, I expect us to take it in turns. But the thing is that where I'm working all week, I can be quite tired, so I need a day off to recover. (Nick, 34 yrs)

None of the participants rejected the notion that fathers should be involved in domestic work, but there were differences in how much should be expected. In contrast to providing hands‐on care, there was less consensus about the importance of domestic work in being a father.

#### 4.1.3 Being a father in public

As well as being a father within the family environment, participants also described the ways they practised being a father beyond the family home, either through employment, or by representing the family to others. As we have shown, Nick's work as an advocate and trainer was an important aspect of his identity and this was echoed by Simon and Darren who also had advocacy roles. These advocacy skills enabled them to represent their family to professionals, such as Nick being “the mother's voice” when his partner was not able to express herself, and Darren writing to the newspapers to complain about the council's bus arrangements for his son.

Darren: …and they printed it. And we got so many responses from people saying, Oh yeah, he should be on the bus, he shouldn't do this. [Name] council are this, [name] council are that. And within two weeks of putting the article in, [name] council wrote back and said, Put him on the bus. (Darren, 53 yrs)

Practising being a father in these ways was not available to all participants and this appeared to limit the scope of what fatherhood could be for them. For example, John worked at a supermarket 2 days a week, pushing trolleys, but when asked if he enjoyed it flatly replied “*No.”* None of Peter, Mustafa or Matt were in paid employment and although they were involved in advocacy services, they used this to advocate on behalf of parents more generally, rather than representing their own family specifically.

#### 4.1.4 Participants' own fathers

Five participants described their own fathers in ways that highlighted the negative ways they had been treated, either through having low expectations, or by being emotionally unavailable, absent or hurtful. For example, Matt explained how the absence of his father meant that his mother had fulfilled both roles:

Interviewer: And you mentioned your dad as well. Is he around?

Matt: No, No, I didn't have a dad. Well we had it, My mum was my dad. (Matt, 47 yrs)

For Matt, his mother fulfilled both roles for him and although Peter was brought up by his grandparents, he also described his relationship with his father as a big issue when his father had promised to visit, but not turned up. Simon's father had been present to bring him up until his parents separated, but his father had led him to feel ashamed of having a learning disability:

Simon: I was ashamed to have a learning disability. Do you know why? My dad – am I OK to talk about that?

Interviewer: Of course

Simon: My dad, my real dad, I don't talk to him anymore. My real dad thought me, the only son, thought I were a let‐down because I've got a learning disability. He thought I weren't man enough. (Simon, 43 yrs)

The experiences with their own fathers offered a version of fatherhood which some participants defined themselves against in their own identities. Nick noted the irony of caring for his father through illness in a way that his own father would not have managed for him, and Simon displayed his motivation to disprove his father's low expectations:

Simon: He told me I would never have kids, I would never have a job, I would never get married or live independent. I'm not married, but I thought three out of four is not bad! (Simon, 43 yrs)

The descriptions of fatherhood presented here reflect the differences between participants in relation to care, housework, and their public presence as a father. Fulfilling public roles through employment was only felt by a minority of participants, whereas all gave examples of direct care for their children, and to a lesser extent, their involvement in domestic duties.

### 4.2 Challenges of fatherhood

Participants also described some of the challenges they had faced as fathers, such as managing the bills and paperwork.

Joe: I think the paying the bills and stuff like that was really hard, because…like any paperwork and all those sorts of things. And I think really what…it's more about the embarrassment, or all those things that people with learning difficulties suffer with all the time, is going and asking for somebody to read private letters, bills. (Joe, 61 yrs)

The embarrassment mentioned by Joe was also compounded by a sense of scrutiny that others might give on his parenting. He went to say that he made sure he ironed his socks and pants because he was worried that “… somebody would come in and say that I was doing it wrong.” Simon described how he had not been prepared for parenthood because he did not know how to cook or pay the rent and had to find help from his family to learn these things.

A second challenge involved the difficulties in caring for children. Mustafa, who was having supervised contact with his children at the time of the interview, referred to his goal of speaking calmly to his children, even when they misbehaved.

Interviewer: What sorts of things are difficult?

Mustafa: Sometimes you need to look after kids when the kids are being naughty. So, try to talk to wife, and kids quietly sometimes. (Mustafa, 34 yrs)

Mustafa was working with his social worker to find better strategies to care for his children, but it is evident in his quote that he was also working on speaking to wife quietly, suggesting he experienced some frustration in his adult relationship as well as with his children. John raised the same issue about feeling stressed in his relationship, where he felt he was doing an unequal amount for his partner than she did for him:

John: “Some days I'm thinking … Don't want to be here any more. It's like I'm a carer. But she treats me I'm a slave and a kid. No one's here this morning, I had a go at her: ‘I'm not a child. I'm not your slave’. I said ‘I don't get paid to be your carer, I want to be your partner.” (John, 38 yrs)

John had considered leaving his relationship, and although his experience was different from Mustafa's, they illustrate the strain experienced in couples meeting each other's needs. Without resolving these difficulties, couples might separate and this had occurred to three of the participants. Simon identified different parenting approaches as one of the sources of disagreement between them:

Simon: I had my mum's point of view. I believe in grounding them and punishing, like being a proper parent. But my ex used to do the opposite. When I'd ground them for a week, they'd be out a couple of days later. Or if I stopped their spending money…she'd do the opposite. (Simon, 43 yrs)

Following their separation, the children lived with her and Simon went for a period of time without seeing the children. The sense of temporary absence came up in different ways, for six participants in total, but all of which interrupted their ability to be fathers.

For example, John told us that his partner's family had not been happy about the couple having a baby and had prevented him from being in the hospital during the birth of his daughter.

John: But I wasn't there when Sarah was born. I wasn't allowed in the hospital

Interviewer: Right. How did you feel about that?

John: Sad. I wanted to be there in the room and see Sarah being born

Interviewer: So why did they stop you going in the room?

John: They didn't want me there. (John, 38 yrs)

Not being allowed to be present for the birth of his daughter continued to be an issue for John and he stated it would be the one thing he would change if he could. Peter was unable to be present at his son's birth, due to his mental illness which had resulted in being admitted to hospital himself. Mental health difficulties were also a factor for Darren, who told us that his post‐natal depression resulted in the temporary separation of his relationship:

Darren: I had a nervous breakdown. And my wife actually left for six months. So, we didn't see each other for six months, because she couldn't help with me, because I was being…but then they found out that what it was, I had post – everyone said that it was the mothers get it, but no one said that the fathers get it.

Interviewer: Oh, post‐natal depression?

Darren: Yeah. And they – no one actually thought that the fathers get it. It turned out that's what I had. (Darren, 53 yrs)

Overall, four of the participants spoke of the ways in which their mental health difficulties impacted on their capacity to parent as fathers and interrupted their opportunities to do so. We use the term interrupted because they did not have final consequences for them as fathers and they continued to seek ways to fulfil a role as a father. For example, Darren was reunited with his partner, and Peter kept in touch with his adopted son through twice yearly letters. Nevertheless, the challenges of interrupted fatherhood meant that participants missed out both on children's development and on their opportunities to be a parent.

All participants described challenges in being a father, whether the stress of being a parent, disagreements with their partner, or negotiating parenting in a separated relationship. Some of these challenges are familiar to any parent, but for six fathers we spoke to, this resulted in what we have described as an interrupted fatherhood which placed additional barriers in way of their desire to parent their children.

### 4.3 Support with fatherhood

Despite challenges, participants also spoke of the help they received from others. Most commonly, this was from female members of the wider family network who maintained a consistently supportive presence in their lives. Despite the “rocky” relationship John had with his partner's parents, he told us about the commitment they received from her sister.

John: Yeah. But Beth's sister…her sister asked her, “Do you still want to be with John,” and she said, “Yes, we love each other.” And then she said, “I'll not stand in your way.”

Interviewer: Right

John: She said, “Don't worry about your mum – mum and dad.” She said, “If that's what you and John want, then I'll stand by you.” (John, 38 yrs)

It is a common finding in the literature that mothers are a key support to parents with learning disabilities and Nick described his mother as “a rock” whose support had meant his daughter to stay in their care. However, participants also referred to other supportive women such as John's sister‐in‐law. Peter told us how his grandmother and father's partner (but not his father) helped him write letters to his adopted son.

Participants told us about professional support for nonparenting related matters such as independent living, finances and housing. For example, Mustafa's social worker supported him to get new accommodation when he had to move out of the family home and we heard about support for mental health difficulties which may have come about from the stress of parenting. Darren described the support he received after having been diagnosed with post‐natal depression.

Interviewer: Where did you get that support from?

Darren: Local GP and all that

Interviewer: Ok. So was that talking therapy, or was it medication, or a bit of both?

Darren: Yeah. Medication, and talk – like a bit of social workers – like – sorry, psychiatrists and all that, yeah. (Darren, 53 yrs)

While the support received by both Mustafa and Darren was well received, they were not focused specifically on their parenting and this was evident for other participants. One exception was given by Nick, who described support from the midwife when his daughter was a baby:

Nick: We had a really good community midwife, who really supported us well. And I think that was because she had a child – she's got a child with a learning disability herself, so she was able to support us in the right way and support us through it.

Interviewer: Right. What kind of things did she do?

Nick: So, she just talked to us about things, arranged meetings, that we all came together as family, other people that supported us together. And supported us really well to understand, this is what needs to happen, and this is what will happen. (Nick, 34 yrs)

Practical support from a person with personal experience was valued by Nick and his use of the collective pronoun “us” suggests that the midwife included both him and his partner in the work. Matt and Mustafa described being part of parenting groups delivered by social care services or advocacy organisations, but when parenting support was delivered at home, this was viewed as focused on the mother and was experienced less positively by participants. Nick went on to say that family support services focused on practical and emotional support to his partner, but did not include him in this. John lived in a supported living environment where staff gave video feedback to his partner Beth, but did not include him.

John: Beth likes it, but…it's like they – they give us a piece of paper, you sign, they film us, they always don't film me, they always film Beth. And I'm thinking, we've signed the form, we should both of us be on there. But it doesn't work like that. (John, 38 yrs)

The focus on the mothers' interactions led to a sense of exclusion for John and this was echoed in interviews with other fathers with different experiences. John's daughter was three at the time of the interview so his experience was relatively recent, but it echoed the views of older participants who described their experiences from years previously, such as Simon's experiences of parenting support when his children were teenagers.

Simon: But I just feel like I didn't have no support. She got all of the support, and I didn't. And I felt like, what do I do? Am I a spare part, or what? (Simon, 43 yrs)

And Matt stated in more categorical language the feeling he had been excluded from support provided to the mother, again, about 10 years previously.

Matt: They support the baby mothers, yeah. But they don't support the father. Don't worry about the father. They kick the father to the curb. (Matt, 47 yrs)

## 5 DISCUSSION

Our study contributes to a small body of knowledge about a group whose voices are rarely heard. Although the participants represented a wide range of experiences, ages and lengths of time as fathers, there is value in how they all related to the single topic of fatherhood. Some of the participants were more articulate than others and like Kaspar and Stenfert Kroese's (2017) study of good motherhood, we found that they produced more data and were represented more often in the findings. Although most of the participants were recruited from advocacy organisations, there were differences between them in their approaches to fatherhood. Nevertheless, it will be important to hear the perspectives of fathers with learning disabilities who are not already engaged with advocacy support. Here, we discuss our findings in relation to three topics: conceptualising fatherhood; interrupted fatherhood; and implications for practice.

### 5.1 Conceptualising fatherhood

Ideas about fatherhood have been transformed over the past 50 years (Dermott, 2008) and this study provides evidence of how fathers with learning disabilities reflect these changes. Although Peck and Stephens (1965) concluded that men with learning disabilities could not be successful fathers, four of the participants we spoke to were living with and bringing up their children. All of the participants we spoke to described fatherhood as including at least some childcare, more so than paid employment, which was evident in Gosden and Kirkland's (2008) study. Although there were different approaches to domestic work, there was evidence of an awareness of equal contributions, and for Simon, a stance of gender equality similar to the father's account in Strike and McConnell (2002). Comparing the accounts from participants against Pleck's framework of involved fathering, participants gave examples of providing hands‐on, direct care for their children and advocating for the family, but future studies may draw on the framework to investigate other dimensions, such as emotional sensitivity, or arranging services, in more detail, especially given that they may differ from mothers in the way they address the parenting identity of fathers (Shewan et al., 2012).

### 5.2 Interrupted fatherhood

Despite their different characteristics, a common experience for six participants was periods of absence from their children, such as being denied attendance at the birth, or needing treatment for post‐natal depression. Rather than treat these episodes as conclusions to their fathering identities, participants continued to attempt to achieve this in some way, such as Peter writing to his adopted son, or Darren accessing support to reintegrate with his family. We are aware that participants may have been motivated to present themselves favourably in the interviews, but the examples given suggest that participants were still committed to the idea of being a father as opportunities allowed. Conceptualising fathers' absence as “interruptions” affords the possibility that fathers with learning disabilities wish to continue in their role even though they may need support to do so.

### 5.3 Implications for practice

We heard examples of participants receiving helpful support from family members, but less so from support services, where Simon, Matt, John and Nick all felt that practical support was focused on the mother. There are three implications for services that we draw here.

The first is that services should recognise and engage with fathers, as well as mothers, in the families they work with. An important consideration in this is that services adopt a whole family approach that recognises the impact of all family members on the well‐being of the child. Such an approach has been promoted in UK national policy for some time (Social Exclusion Task Force, 2007) and focuses on recognising the strengths and needs of both parents. Even if the father is not currently known to be present in the child's life, our findings suggest that this may be due to an interruption in the father's involvement rather than indicating he has no involvement at all. It may actually be a point at which he is in particular need of support.

Previous work by Tarleton et al. (2006) found that when good support is provided early, this can enable parents with learning disabilities to care successfully for their children. There is good evidence that providing support in the home is more effective than in group settings (Wade et al., 2008), but the participants we spoke to told us that this frequently focused on the mother and our findings support recommendations to include fathers in parenting support services. Conversations with fathers could include managing the stress of parenting tasks (such as managing children's misbehaviour), asking about fathers' mental health, and identifying tensions in the couple relationship where fathers may have strong feelings about parenting styles, expectations of housework, or find it difficult to manage disagreements. When there is a risk to the child, it will be appropriate not to involve the father, but on other occasions, services should ensure that fathers are included in home visits so that early support can be given effectively to the whole family.

### 5.4 Conclusion

Although the literature on parenting with a learning disability is now extensive, this article provides a new perspective by focusing specifically on fathers. Eight fathers with learning disabilities described the different ways that they were involved in their children's lives and the challenges they faced. Some participants managed these with their partners, or with family, but it was common to feel left out by services. Without the right support, the challenges of being a father may interrupt their involvement and this raises implications for parenting and learning disability services. By taking a whole family approach and engaging fathers during home visits, fathers with learning disabilities can be supported to provide good care to their children.

## Data Availability

Research data are not shared.
